# Thermal ablation of a confluent lesion in the porcine kidney with magnetic resonance guided high intensity focused ultrasound

**DOI:** 10.1186/2050-5736-3-S1-P76

**Published:** 2015-06-30

**Authors:** Johanna van Breugel, Joost Wijlemans, Martijn de Greef, Gerald Schubert, Maurice van den Bosch, Chrit Moonen, Mario Ries

**Affiliations:** 1 University Medical Center Utrecht, Utrecht, Netherlands; 2 Philips Healthcare, Vantaa, Finland

## Background/introduction

Since approximately 1.6 percent of men and women will be diagnosed with kidney and renal pelvis cancer during their lifetime, there is a growing interest in non-invasive kidney sparing therapy for renal cancer. As a consequence, several patient studies investigated the feasibility of high intensity focused ultrasound (HIFU) for the thermal ablation of renal masses. The majority of these studies used either a hand-held extracorporeal ultrasound transducer with ultrasound imaging for guidance or a laparoscopic approach. Drawbacks of these techniques are the lack of respiratory motion compensation, no means to observe the energy deposition in real time, the complexity of the probe positioning, and the risks of bleeding and tumor spillage. Alternatively, recent preclinical studies have demonstrated the feasibility of magnetic resonance guided high intensity focused ultrasound (MR-HIFU) interventions on the kidney with respect to motion compensated real-time thermometry and acoustic energy delivery. Here, we extend this prior work to investigate in an animal study if MR-HIFU can deliver a reliable confluent volumetric lesion in the renal cortex in a clinically relevant time-frame.

## Methods

**Figure 1:**
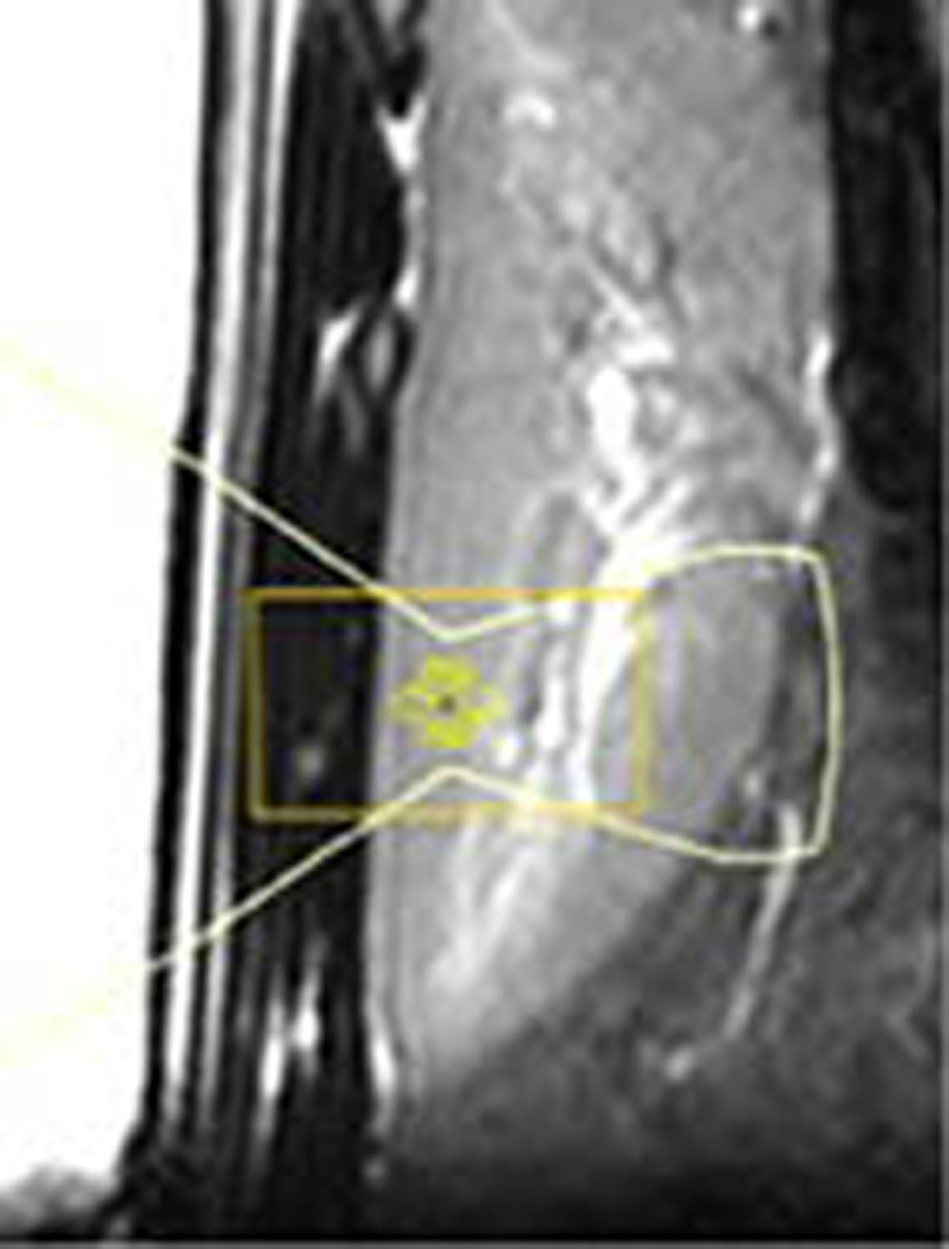
Planning of 7 treatment cells in the cortex of the kidney

An anesthetized Dalland land pig was placed on its right side on a clinical Sonalleve MR-HIFU therapy system, which is integrated with a 1.5T Achieva MRI (Philips Healthcare) with minor modifications. Both acoustic energy delivery and MR-thermometry were respiratory gated (3mm gating window, ~70% duty cycle) and active surface cooling was employed to prevent undesired near-field damage. A honeycomb pattern of seven ablation cells (12-17s, 450W acoustic power, 4x4x10 mm3) was positioned in the cortex of the kidney as shown in figure 1. The therapeutic endpoint was evaluated non-invasively at the end of the intervention. Hereto, lethal thermal dose estimates based on MR-thermometry and a non-perfused volume (NPV) measurement using dynamic contrast enhanced T1-weighted MRI (DCE-MRI) were performed. Subsequently, the animal was euthanized and the extent of the induced necrosis examined using a cellular viability staining (nicotinamide adenine dinucleotide, NADH).

## Results and conclusions

**Figure 2:**
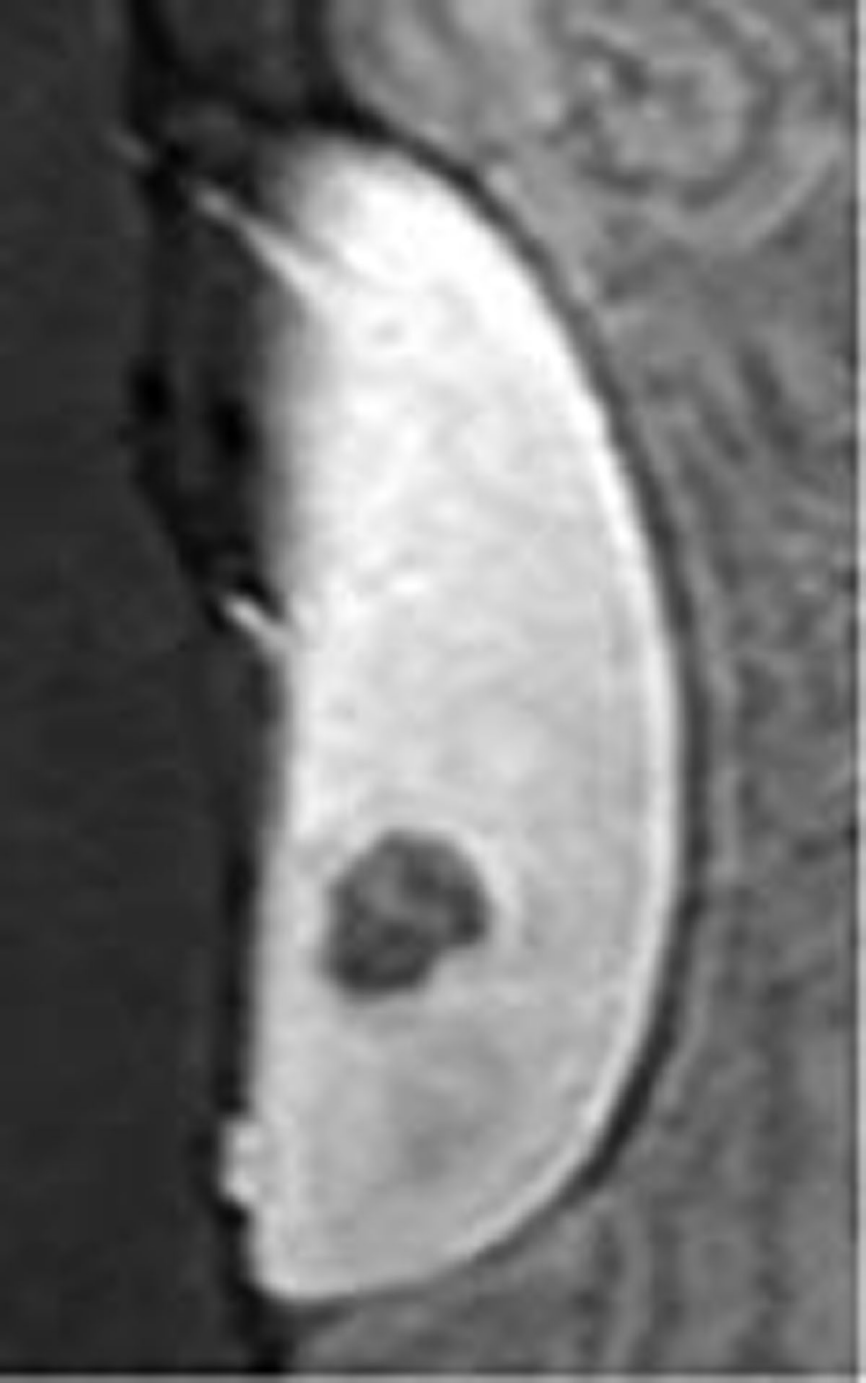
Contrast enhanced MR scan shows a non-perfused volume in the kidney

**Figure 3:**
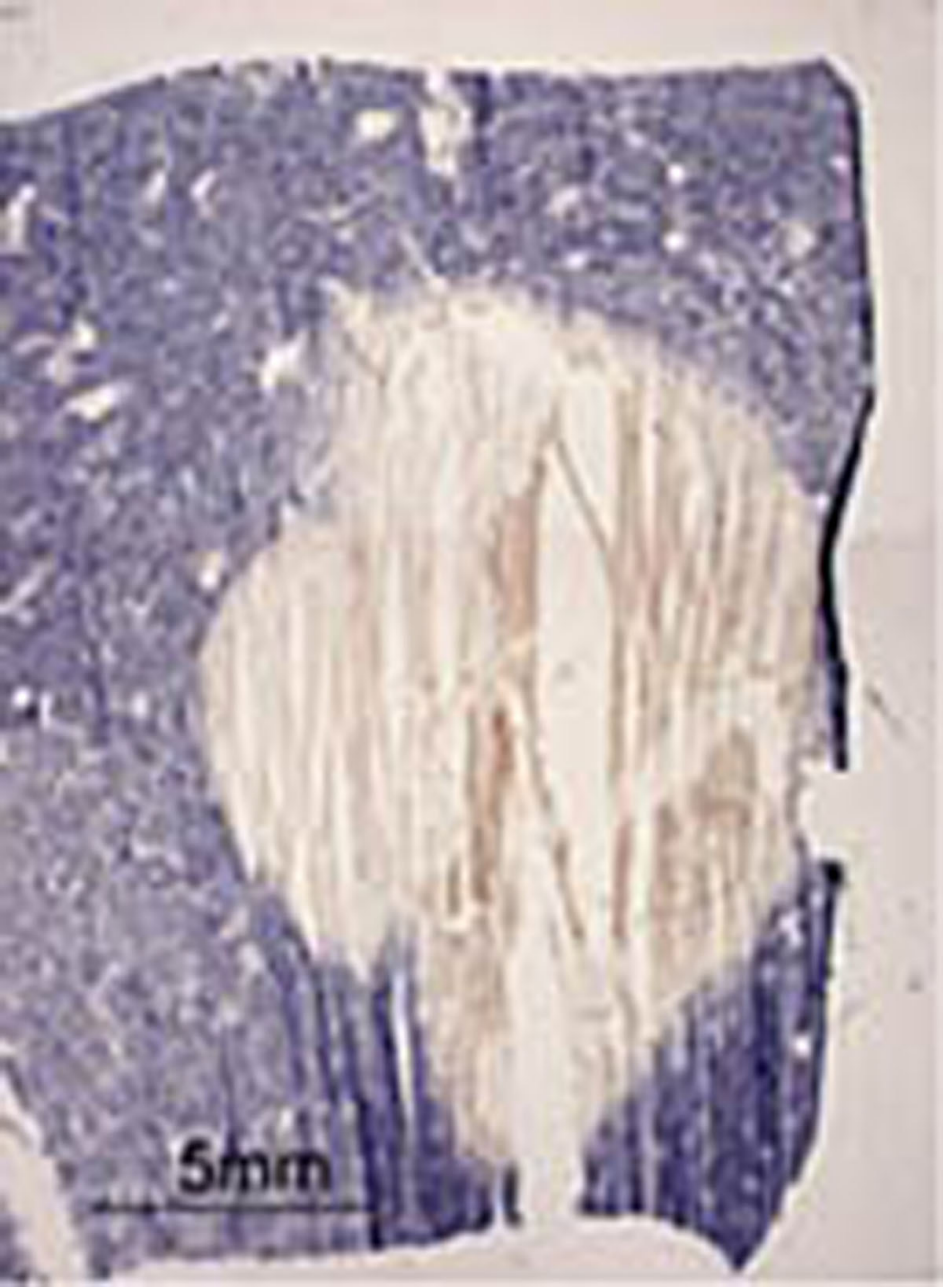
NADH staining shows a non-viable area (yellowish) within viable kidney tissue (blue)

All ablation cells reached peak temperatures > 80°C during the sonications, resulting in a lethal thermal dose over the entire delineated target area. DCE-MRI displayed a confluent non-perfused volume within the cortex and partly within the medulla with a volume of ±2 ml as shown in figure 2. NADH staining reconfirmed a confluent non-viable volume of approximately (12 x 16 x 10 mm3) (Figure 3). No undesired tissue damage in adjacent areas has been observed. These first results indicate that current MR-guided clinical HIFU equipment might be suitable for non invasive therapy of renal masses. Future work will need to demonstrate the reproducibility of the findings and investigate potential adverse effects (cutaneous and subcutaneous damage) as a preparation for a clinical study.

